# Comparative Analysis of Biological Properties of Large-Scale Expanded Adult Neural Crest-Derived Stem Cells Isolated from Human Hair Follicle and Skin Dermis

**DOI:** 10.1155/2019/9640790

**Published:** 2019-02-19

**Authors:** Roman G. Vasyliev, Olga S. Gubar, Inna M. Gordiienko, Larisa S. Litvinova, Anzhela E. Rodnichenko, Valeria V. Shupletsova, Alona V. Zlatska, Kristina A. Yurova, Natalia M. Todosenko, Veronika E. Khadzhynova, Mariia V. Shulha, Svitlana N. Novikova, Dmytro O. Zubov

**Affiliations:** ^1^Department of Genetic Diagnostics, State Institute of Genetic and Regenerative Medicine NAMS of Ukraine, 67 Vyshgorodska Str., Kyiv 04114, Ukraine; ^2^Biotechnology Laboratory ilaya.regeneration, Medical Company ilaya^®^, 9 I. Kramskogo Str., Kyiv 03115, Ukraine; ^3^Department of Functional Genomics, Institute of Molecular Biology and Genetics NAS of Ukraine, 150 Zabolotnogo Str., Kyiv 03143, Ukraine; ^4^Department of Molecular and Cellular Pathobiology, R.E. Kavetsky Institute of Experimental Pathology, Oncology and Radiobiology NAS of Ukraine, 45 Vasylkivska Str., Kyiv 03022, Ukraine; ^5^Basic Laboratory of Immunology and Cell Biotechnologies, Immanuel Kant Baltic Federal University, Kaliningrad 236041, Russia; ^6^Department of Cell and Tissue Technologies, State Institute of Genetic and Regenerative Medicine NAMS of Ukraine, 67 Vyshgorodska Str., Kyiv 04114, Ukraine

## Abstract

**Introduction:**

The adult neural crest-derived stem cells (NCSCs) have significant perspectives for use in regenerative medicine. The most attractive sources for adult NCSC isolation are the hair follicles (HF) and skin dermis (SD) because of easy access and minimally invasive biopsy. The aim of this study was to compare the biological properties of HF- and SD-derived NCSCs after their large-scale expansion.

**Methods:**

The conventional explant method was used to obtain HF NCSCs. For the isolation of SD NCSCs, a new combined technique consisting of preplating and subsequent culturing in 3D blood plasma-derived fibrin hydrogel was applied. The studied cells were characterized by flow cytometry, ICC, qPCR, Bio-Plex multiplex assay, and directed multilineage differentiation assays.

**Results:**

We have obtained both adult SD and HF NCSCs from each skin sample (*n* = 5). Adult SD and HF NCSCs were positive for key neural crest markers: SOX10, P75 (CD271), NESTIN, SOX2, and CD349. SD NCSCs showed a higher growth rate during the large-scale expansion compared to HF NCSCs (*p* < 0.01). Final population of SD NCSCs also contained more clonogenic cells (*p* < 0.01) and SOX10^+^, CD271^+^, CD105^+^, CD140a^+^, CD146^+^, CD349^+^ cells (*p* < 0.01). Both HF and SD NCSCs had similar gene expression profiling and produced growth factors, but some quantitative differences were detected. Adult HF and SD NCSCs were able to undergo directed differentiation into neurons, Schwann cells, adipocytes, and osteoblasts.

**Conclusion:**

The HF and SD are suitable sources for large-scale manufacturing of adult NCSCs with similar biological properties. We demonstrated that the NCSC population from SD was homogenous and displayed significantly higher growth rate than HF NCSCs. Moreover, SD NCSC isolation is cheaper, easier, and minimally time-consuming method.

## 1. Introduction

The neural crest (NC) is a transient structure appearing during the embryonic development of *Vertebrates* [1] that is formed on the border between the somatic ectoderm and the neural plate [2]. The Canadian scientist Brain Hall assumed that NC is a fourth embryonic layer taking into consideration its role in *Vertebrates* ontogenesis and phylogenesis [3]. This concept is becoming increasingly common in the scientific community. After their specification, the NC cells undergo delamination and distant migration to target tissues and organs. Numerous cell types and tissues are derived from NC, including the bone, cartilage, and connective tissue in the head and neck region, neurons and glia of the peripheral nervous system, melanocytes, endothelial, and stromal (keratocytes) corneal cells, and some endocrine cells of the APUD system [4]. There are several domains within NC, among which the cells of the cranial neural crest possess the most wide-ranging potential for multilineage differentiation. They give rise to ectomesenchyme (i.e., different mesenchymal cell types, like adipocytes, osteoblasts, and chondrocytes), melanocytes, neurons, and glia of the peripheral nervous system [4].

Such a wide potential to multilineage differentiation implies the existence of multipotent stem cells. The presence of NC stem cells in mammals was first shown in 1992 at premigratory/early migratory stage [5]. Since 1997, neural crest-derived multipotent stem cells (NCSCs) have been identified and isolated from a number of tissues and organs of mammals at later fetal and postnatal stages of development: the small intestine [6], dorsal roots of the spinal cord [7], the bulge region [8] and the dermal papilla [9] of the hair follicle (HF), skin dermis (SD) [10], adipose tissue [11], bone marrow [12], palate [13], gingiva [14], nasal mucosa [15], dental pulp [16], periodontal ligament [17], heart [18], corneal [19] and iris [20] stroma, etc. The history of discovery and study of adult NCSCs, their tissue sources, and biological properties are summarized in several recent reviews [21, 22]. Adult NCSCs have the ability to undergo directed differentiation into adipocytes, osteoblasts, chondrocytes, melanocytes, neurons, and Schwann cells [21, 22]. Moreover, NC cells possess the plasticity of the *HOX* code, which determines the positional information of the cells in the body. This property allows the NC cells, after transplantation into the damaged tissue site, to modify their original *HOX* code and acquire the characteristic of host tissue *HOX* code. Importantly, damaged tissue can have a non-NC origin and be arisen from other embryonic layers (e.g., the mesoderm). This phenomenon was first described for the mandibular skeletal progenitor cells, which have NC origin, after their transplantation into the bone defect of the *tibia* (mesodermal origin) [23]. NC-derived nasal chondrocytes after transplantation into the defect of articular cartilage of the knee (mesodermal origin) also demonstrated *HOX* code plasticity [24]. It is likely that *HOX* code plasticity ensures the correct structural and functional integration of the transplanted NC cells into the host tissue of other embryonic origin. In addition, under certain experimental conditions *in vitro* and *in vivo*, adult NCSCs give rise to CNS-specific cells, including neural stem cells [25], oligodendrocytes [26], astrocytes [27, 28], and dopaminergic neurons [29, 30]. Also in the skin wound model, it was shown that adult NCSCs regulate the process of reparative regeneration after injury and their elimination leads to the aberrant skin repair [31].

Adult cultured NCSC therapeutic application led to the restoration of bone [32] and cartilage defects [33] and damaged peripheral nerves [34, 35] and spinal cord [36, 37]. Moreover, native and/or predifferentiated adult NCSCs were able to undergo structural and functional integration into the brain after transplantation [38, 39]. Thus, due to the substantial multilineage differentiation potential and *HOX* code plasticity, adult NCSCs are attractive candidates for application in regenerative medicine [40], especially in the field of neurology and neurosurgery [41].

Adult NCSCs are represented by a minor heterogeneous population of cells in tissues and organs. Therefore, selective isolation of adult NCSCs and their effective large-scale expansion are important technical issues for the successful applications for regenerative medicine purposes. Various techniques were applied for the selective isolation of adult NCSCs: fluorescence-activated cell sorting [6, 42], selective culturing conditions for growth as neurosphere-like structures [42, 43], explant technique [44, 45], etc.

Promising sources for the isolation of adult NCSCs are the SD and HF due to the come-at-able and minimally invasive biopsy procedure. Explant technique is traditionally used for isolation and large-scale expansion of adult NCSCs from human hair follicles [44, 45]. However, the main and significant drawbacks of explant method are high frequency of explant detachment, spontaneous outgrowth of keratinocytes from HF explants, and long-term primary culturing step to obtain sufficient therapeutic cell number for subsequent expansion step [44, 45]. The originally described and widely used method for isolation and expansion of SD NCSCs is based on their ability to form neurosphere-like structures in a specific serum-free growth medium and ultra-low attachment culture dishes [42, 43]. On the one hand, this method allows obtaining selective growth of NCSCs from a heterogeneous cell population containing cells of mesodermal origin. From the other hand, the disadvantages of this method are the relatively low growth rate of NCSCs and the high cost of cell culturing due to the use of special ultra-low attachment culture vessels, high concentrations of recombinant growth factors, and synthetic growth supplements for replacement of the fetal bovine serum. Thus, the scan of new ways to optimize NCSC isolation and large-scale expansion is still a perspective vector in regenerative medicine. The aim of this study was to compare the biological properties of human adult HF NCSCs obtained by explants method and SD NCSC isolated by our alternative method through preplating and fibrin hydrogel after their large-scale expansion.

## 2. Materials and Methods

All procedures related to obtaining human skin biopsies, cell isolation, and culturing were performed with written informed donor consent and in accordance with the laws of Ukraine. The study protocol was approved by the Bioethics Committee of the State Institute of Genetic and Regenerative Medicine NAMS of Ukraine (Kyiv, Ukraine). The cell culturing was carried out in the GMP/GTP-compliant biotechnological laboratory *ilaya.regeneration* (License to operate the banks of human cord blood, other tissues, and cells; issued by the Ministry of Health of Ukraine AE no. 186342 from 11.07.2013).

The skin specimens were obtained from 5 healthy male donors aged 20 to 28 years.

### 2.1. Donor and Cell Culture Screening Tests

Peripheral blood samples from donors (ELISA, PCR) and operated cell cultures (PCR) were screened for HIV½, HBV, HCV, HSV½, CMV, EBV, *Treponema pallidum, Mycoplasma hominis*, and *Mycoplasma genitalium* infections. The cell cultures were also tested for potential mycoplasma contamination by PCR with Mycoplasma Test Kit I (AppliChem, Germany). The normal karyotype of cultured cells was confirmed by GTG-banding method at the final passage.

### 2.2. Isolation and Culturing of Adult NCSCs from Human Hair Follicle and Skin Dermis

The cultures of HF NCSCs were obtained according to explant method by Sieber-Blum et al. [8] in our modification for human samples [45]. Briefly, two skin specimens per donor were excised from the scalp zone under local anesthesia by dermal punch knife (Dermo-Punch ø 4 mm, Sterilab, Italy). Skin specimens were incubated overnight at 4°C in Dispase II solution (0.4 U/ml; Sigma-Aldrich, USA). Then an epidermis was carefully removed from the dermis and HFs were isolated under the control of Stemi 2000 stereomicroscope (Carl Zeiss, Germany). The HFs were explanted on a thin layer of collagen gel in two Petri dishes ø 35 mm (SPL, Korea) and incubated for 40 min at 37°C in a multigas incubator (Binder CB 210, Germany) in an atmosphere of 5% CO_2_ and 5% O_2_ and a saturating humidity of 97% for attachment before adding complete growth medium.

For NCSC isolation, cells from SD (after overnight incubation at 4°C in 2 U/ml Dispase II solution and epidermis removal) were minced and digested in 0.1% collagenase/0.1% pronase mixed solution (all: Sigma-Aldrich, USA) for 3 h. Obtained cell suspension was centrifuged for 10 min at 800 g (Eppendorf 5804R, Germany). Cell pellet was resuspended in 10 ml complete growth medium and seeded for preplating into standard cell culture plastic Petri dish ø 100 mm (SPL, Korea) for 48 h. For preplating, the following growth medium was used: *α*MEM w/o nucleosides (Gibco, UK), 10% FBS PharmaGrade (Sigma-Aldrich, USA), 2 mM stable *L*-glutamine (BioWest, France), and 1% antibiotic/antimycotic solution (BioWest, France). After preplating, a nonadherent cell population was collected and centrifuged at 600 g for 5 min (Eppendorf 5804R, Germany). Cell pellet was resuspended in 4.5 ml platelet poor blood plasma obtained from the same donor. Then 0.5 ml of a mixture consisting of 0.45 ml of blood serum (from the same donor) and 50 *μ*l 10% CaCl_2_ solution was added to the cell suspension. The resulting mixture was quickly transferred into the T25 flask (SPL, Korea) and placed for 15 min in an incubator for hydrogel polymerization. After the formation of 3D fibrin hydrogel, 5 ml of complete growth medium was added to the flask. The cell isolation from the fibrin hydrogel for subculturing was performed by incubation with 0.1% pronase (Sigma-Aldrich, USA) for 10 min. The resulting cell suspension was diluted 5× with PBS (Sigma-Aldrich, USA) and centrifuged for 5 min at 600 g (Eppendorf 5804R, Germany) to wash out the enzyme remnants. The pellet was resuspended and washed one more time with PBS (Sigma-Aldrich, USA). The cells were resuspended in a growth medium and cultured in collagen-coated culture flasks.

After primary culturing, the cells were subcultured with 0.01% trypsin in 0.53 mM Na_2_EDTA solution (Sigma-Aldrich, USA) and seeded into collagen-coated culture flasks (T25 flask, SPL, Korea; or 5-layer multiflask, Corning, USA) with plating density 1000 cells per cm^2^.

The cells were cultured in a multigas incubator CB 210 (Binder, Germany) at 37°C with 5% CO_2_ and 5% O_2_ and a saturating humidity. For the expansion of adult HF and SD NCSCs, the following growth medium was used: *α*MEM w/o nucleosides (Gibco, UK), 5% FBS PharmaGrade (Sigma-Aldrich, USA), 5 ng/ml bFGF (Gibco, UK), 10 ng/ml EGF (Gibco, UK), 1% ITS supplement (Gibco, UK), 2 mM stable *L-*glutamine (BioWest, France), 1% antibiotic/antimycotic solution (BioWest, France), and 2 U/ml heparin (Indar, Ukraine).

To assess the growth rate by cell total yield number after culturing of adult HF and SD NCSCs at P3 (2D vs. 3D cultures), 10^4^ cells were seeded per Petri dish ø 35 mm (SPL, Korea) for 2D (*n* = 5) or 3D within fibrin hydrogel (*n* = 5) culturing over 7 days.

To assess the ability of the NCSCs to grow as floating neurosphere-like structures, 1000 cells per well were seeded into the ultra-low attachment 24-well plate (Corning, USA) in the following serum-free growth medium: neurobasal medium (Gibco, UK), 2% B27 supplement (Gibco, UK), 1% N2 supplement (Gibco, UK), 20 ng/ml bFGF (Gibco, UK), 40 ng/ml EGF (Gibco, UK), 2 mM stable *L*-glutamine (BioWest, France), and 1% antibiotic/antimycotic solution (BioWest, France).

The cell population doubling number (PDN) and cell population doubling time (PDT) were calculated according to the following standard formulas [46]:

(1) $$\begin{array}{c} \text{PDT}=\frac{T}{3.31\;\mathrm{lg}}\left(\frac{X_{k}}{X_{0}}\right),\\ \text{PDN}=3.31\;\mathrm{lg}\left(\frac{X_{k}}{X_{0}}\right), \end{array}$$

where ${\underset{¯}{X}}_{\underset{¯}{k}}$ is the number of obtained cells; *X*_0_ is the number of plated cells; *T* is the cell culture time.

### 2.3. CFU Assay and Self-Renewal Capacity

To assess the clonogenic potential, the NCSCs at the last passage (P3) were seeded in the number of 100-300 cells on collagen-coated ø 100 mm Petri dishes (SPL, Korea) in growth medium supplemented with 20% FBS PharmaGrade (Sigma-Aldrich, USA) and cultured for 14 days. The cells were either fixed and stained for colony-forming unit (CFU) assay or subcloned for self-renewal assay. The effectiveness of colony formation (plating efficiency, PE) was calculated using the standard formula [46]:

(2) $$\begin{array}{c} \text{PE},\%=\left(\frac{\text{no}.\text{ colonies counted}}{\text{no}.\text{ cells inoculated}}\right)\times 100. \end{array}$$

The transfer of the clonal colony (subcloning) was executed using cloning cylinders (Sigma-Aldrich, USA). The cells were cultured for 14 days, fixed, and stained for analysis for the formation of secondary CFUs.

### 2.4. Immunocytochemical Analysis

For immunocytochemical analysis, the cells were fixed for 20 min with cold 4% paraformaldehyde, permeabilized for intracellular staining for 20 min with 0.1% Triton X-100, 0.2% Tween 20 in PBS, and blocked for 30 min in PBS with 1% BSA, 5% FBS (all: Sigma-Aldrich, USA). The slides were incubated with primary antibodies overnight at 4°C, washed three times with PBS, incubated with secondary antibodies for 1 hour at room temperature, washed twice with PBS, stained with Hoechst 33342 (Sigma-Aldrich, USA) for 5 min, washed twice with PBS (Sigma-Aldrich, USA), and mounted in Mowiol mounting medium (Sigma-Aldrich, USA). Detailed information on antibodies is presented in Table S1.

### 2.5. Flow Cytometry Analysis of Cell Surface and Intracellular Marker Expression

The adult NCSC immunophenotype was assessed at the final passage (P3) with fluorochrome-conjugated mouse monoclonal antibodies to CD34, CD45 CD56, CD73, CD90, CD105, CD117, CD140a, CD140b, CD146, CD166, CD271, and NESTIN in accordance with the manufacturer's instructions (BD Biosciences, USA). For detection of SOX10 and CD349 (FRIZZLED-9) primary unconjugated antibodies and secondary Alexa-647-conjugated monoclonal antibodies (Thermo Fisher, USA) were used. Detailed information on antibodies is presented in Table S1. For intracellular staining, the cells were fixed with Cytofix solution, permeabilized, and stained in PhosFlow buffer (all: BD Biosciences, USA) according to the manufacturer's instructions. The assay was performed using a flow cytometer-sorter BD FACSAria™ I and BD FACS Diva 6.1 software (BD Biosciences, USA). Histogram generation was performed with Cyflogic v.1.2.1 software (CyFlo Ltd., USA).

### 2.6. qPCR

Total RNA was isolated from 10^6^ NCSCs using NucleoZOL (MACHEREY-NAGEL GmbH & Co. KG, Germany) according to manufacturer's protocol. The RNA quality and concentration were determined with a spectrophotometer NanoDrop™ 1000 (Thermo Scientific, USA). 2 *μ*g of isolated RNA was reverse transcribed to cDNA using RevertAid First Strand cDNA Synthesis kit (Thermo Scientific, USA). qPCR was performed with a 7500 Real-Time PCR System (Applied Biosystems, CA, USA) using 5× HOT FIREPol^®^ EvaGreen^®^ qPCR Mix Plus (ROX) (Solis BioDyne, Estonia). The Applied Biosystems 7500 system software (V. 1.3.1) was used for data analysis. The primer sequences are listed in Table S2. The following PCR cycling conditions were applied: 10 min at 95°C, 40 cycles of 10 s at 95°C, and 40 min at 60°C. The expression level of TATA box-binding protein (TBP) was used as the internal control. Ct values for target genes were normalised against the Ct value of TBP at the same threshold level. The relative quantification (comparative Ct (ΔΔCt) method) was used to compare the expression levels of the target genes with the internal control. Dissociation curve analysis was performed after every run to check the primer specificity. Results were presented in relative units. For all cell types, reaction was performed three times (each gene in triplicate). GraphPad Prism 4 (GraphPad Software, USA) and MS Excel were used for statistical analysis and graphic data presentation. Box blots show cumulative data of relative mRNA expression in HF NCSC and SD NCSC cell cultures isolated from five donors. In box plots, bars mean maximum and minimum values, the line within the rectangle shows the median, and the top and bottom of the rectangle represent the third and first quartile, respectively.

### 2.7. Production of Cytokines, Chemokines, and Growth Factors by HF and SD NCSCs

The content of targeted proteins in cell culture supernatants after 24 h cultivation in serum-free medium was measured by continuous-flow fluorometry with a double-beam laser automatic analyzer Bio-Plex Protein Assay System (Bio-Rad, USA) using commercial kits (Bio-Plex Pro 27-Plex and 21-Plex Assay, Bio-Rad, USA) in accordance with the manufacturer's instructions.

### 2.8. Directed Multilineage Differentiation Assay

Our protocol of directed multilineage differentiation assay for human adult NCSCs was described earlier in details [45]. Briefly, we used the following differentiation media and adhesive substrates: adipogenic differentiation medium: DMEM high glucose (4.5 g/l) (BioWest, France) supplemented with 10% FBS, 1 *μ*M dexamethasone, 50 *μ*M indomethacine, 250 *μ*M isobutylmethylxanthine, 5 *μ*g/ml insulin (all: Sigma-Aldrich, USA), and 5% horse serum (BioWest, France); osteogenic differentiation medium: DMEM low glucose (1.0 g/l) (BioWest, France) supplemented with 10% FBS, 100 nM dexamethasone, 10 mM *β*-glycerophosphate, and 50 *μ*g/ml ascorbate-2-phosphate (all: Sigma-Aldrich, USA); neuronal differentiation medium: neurobasal medium (Gibco, UK), 2% B27 supplement (Gibco, UK), 1% N2 supplement (Gibco, UK), 5 *μ*M ec32 synthetic retinoid (AMSBIO, UK), 1 *μ*M forskolin (Sigma-Aldrich, USA), 20 ng/ml NGF (PeproTech, USA), 20 ng/ml BDNF (PeproTech, USA), 20 ng/ml GDNF (PeproTech, USA), and 20 ng/ml IGF (Gibco, UK); glial differentiation medium: neurobasal (Gibco, UK) and DMEM:F12 (Gibco, UK) media in a ratio 1 : 1, 2% B27 supplement (Gibco, UK), 1% N2 supplement (Gibco, UK), 1 *μ*M ec32 synthetic retinoid (AMSBIO, UK), 5 *μ*M forskolin (Sigma-Aldrich, USA), 20 ng/ml Neuregulin-1 (Gibco, UK), 10 ng/ml PDGF-BB (PeproTech, USA), and 20 ng/ml IGF (Gibco, UK).

Adipogenic and osteogenic differentiation assays of adult HF and SD NCSCs were carried out on collagen substrate. Neuronal and glial differentiation tests were done on poly-*L*-lysine- and laminin-coated surfaces.

### 2.9. Cytochemistry

For CFU staining, the cell colonies were fixed for 20 min with cold ethanol, washed with water, and stained with azure-eosin by Romanowsky-Giemsa method (all: Makrokhem, Ukraine) for 20 min. To confirm the osteogenic and adipogenic differentiation, the cells were fixed for 20 min in 10% formalin (Makrokhem, Ukraine), washed with PBS (Sigma-Aldrich, USA), and stained for 20 min with 2% solution of Alizarin Red S (pH 4.1; for detecting calcified extracellular matrix deposits) or 0.5% solution of Oil Red O (for staining of neutral lipids) and azure-eosin for Romanowsky-Giemsa counterstaining, respectively (all: Sigma-Aldrich, USA). For confirmation of osteogenic differentiation the detection of alkaline phosphatase (ALP) by BCIP^®^/NBT Liquid Substrate System (Sigma-Aldrich, USA) was also performed. The cell viability within fibrin hydrogels was confirmed by the combined staining with fluorescein diacetate (FDA, 2 *μ*g/ml)/propidium iodide (PI, 2 *μ*g/ml) fluorescent dyes (Sigma-Aldrich, USA). The cells were stained with FDA/PI for 5 min at RT, then washed twice with DPBS and mixed with plasma for fibrin hydrogel polymerization in an incubator at 37°C.

### 2.10. Microscopy

Microscopy examinations of live cells, cell cultures, and cytological slides were carried out with inverted AxioObserver A1 microscope equipped with an AxioCam ERc 5s digital camera and ZEN 2012 software. Confocal microscopy examinations were performed with a Zeiss LSM 510 META microscope with LSM Image Browser software (all: Carl Zeiss, Germany).

### 2.11. Statistics

The data are presented as mean ± standard deviation (M ± SD). The Kolmogorov-Smirnov test was used to assess the normality of variable distribution. Student's *t*-test and the Mann-Whitney *U* test were used to determine the statistical significance of differences. The significance level was set at *p* < 0.05.

## 3. Results

### 3.1. Adult NCSCs Derived from Hair Follicles and Skin Dermis Express the Same Key Markers

The HF and SD are well-known sources of adult NCSCs in humans and other mammalians. We used two fundamentally different techniques for HF NCSC and SD NCSC isolation, culturing, and expansion. The explant technique in combination with a specific growth medium was applied to obtain adult HF NCSCs. Specific growth medium provides suitable conditions for the preferable growth of the target NCSC population comparing to keratinocytes and melanocytes, which can also be obtained from the same HF explant. It should be noted that the specific growth medium used in our study is not selective and does not exclude the possibility of keratinocyte outgrowth from the HF explant. The homogenous NCSC population is obtained by differential trypsinization when passaging the primary cell culture, as described earlier [45].

Fibroblasts are the main cell type in SD that possesses high adhesiveness to culture plastic. This property allows them to attach to the culture dishes without any additional coating by adhesive substrates. At the same time, adult NCSCs have less adhesive properties and require the use of different adhesive proteins for their successful culturing, like fibronectin, laminin, or collagen. Based on differences in adhesiveness of fibroblasts and NCSCs, we performed the preplating technique for 48 h, followed by the transfer of unattached cells into the specific adhesive microenvironment. For the latter, we used 3D blood plasma-derived fibrin hydrogel (PDFH), which is permissive for adult NCSC adhesion and it supports their growth in 3D cell culture system [47, 48].

**Figure 1:**
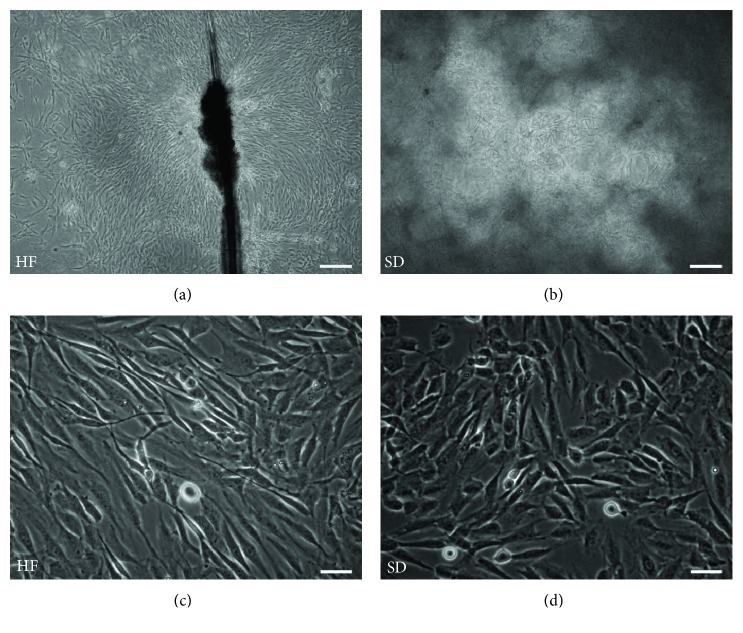
Cell morphology of adult NCSCs isolated from hair follicle and skin dermis. (a) Hair follicle explant demonstrates net outgrowth of HF NCSCs; (b) SD NCSCs within 3D fibrin hydrogel; (c) fibroblast-like morphology of HF NCSCs at passage 1; (d) polygonal morphology of SD NCSCs at passage 1. Phase-contrast microscopy. Scale bar: (a, b) 200 *μ*m; (c, d) 50 *μ*m.

Representative images of adult NCSC primary cultures from the explanted HF and from dissociated SD in 3D fibrin hydrogel are presented in Figures 1(a) and 1(b). It should be noted that adult HF and SD NCSCs had slightly different morphology when cultured under identical conditions (same growth medium and 2D culture on collagen-coated culture plastic at P1): adult SD NCSCs possess compact polygonal morphology (Figure 1(d)), whereas adult HF NCSCs demonstrated more elongated fibroblast-like morphology (Figure 1(c)).

**Figure 2:**
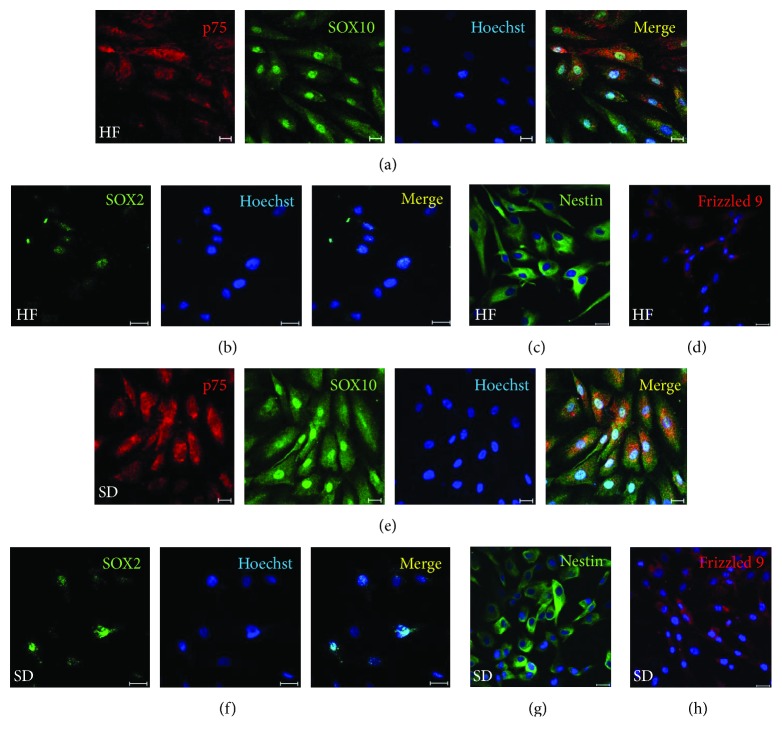
Cells isolated from hair follicle and skin dermis express the key markers of NCSCs. (a–d) HF NCSC at passage 1; (e–h) SD NCSCs at passage 1. Results of immunocytochemical analysis. (a, e) p75/CD271 (red), SOX10 (green). (b, f) SOX2 (green). (c, g) NESTIN (green). (d, h) FRIZZLED-9/CD349 (red). The nuclei were stained with Hoechst 33342 dye. Scale bar: 20 *μ*m.

To confirm the NC origin, we performed an immunocytochemical analysis for NC marker expression by the cells isolated from the HF and SD after the primary culture. Most cells within both populations of adult HF and SD NCSCs expressed SOX10, CD271 (P75, LNGFR), NESTIN, SOX2, and CD349 (FRIZZLED-9) at the protein level. These are the minimal essential criteria for NCSC identity (Figure 2).

Thus, the cell isolation procedure from the SD and HF using different methodological techniques allows obtaining primary NCSC cultures based on the expression pattern of key NC markers.

### 3.2. Isolation and Expansion Efficiency of Adult NCSCs from Hair Follicle and Skin Dermis

The basic growth rate data and the cell yield values during large-scale expansion of adult SD and HF NCSCs from the primary culture up to P3 are presented in Table 1. It should be noted that it is difficult to correctly compare the two methods of adult NCSC isolation (especially regarding the primary culture step) due to significant internal differences between the methods (explant technique vs. enzymatic isolation; 2D vs. 3D conditions of primary cultures) and due to the lack of data about the exact number of adult NCSCs within hair follicle and skin dermis biopsies.

In our study, the time for obtaining primary cell cultures both from HF explants and from dermis using enzymatic isolation, preplating, and subsequent cell culturing in 3D fibrin hydrogel was the same—about 14 days. However, these two methods showed significant differences in total cell yield number at the primary cell culture step. The total cell yield number after the primary culture was 0.165 ± 0.042 × 10^6^ for HF NCSCs vs. 2.349 ± 0.629 × 10^6^ for SD NCSCs (*p* < 0.001) (Table 1). The SD NCSC number at P0 was already enough for seeding in a 5-layer multiflask with growth area 875 cm^2^ considering that plating density for subsequent passages of adult NCSCs is 1000 cells per 1 cm^2^. In contrast, HF NCSCs require an additional passage before proceeding to the large-scale expansion in the 5-layer multiflask. Due to the use of different culture vessels at P1 for the growth of HF NCSCs and SD NCSCs (T25 flask *vs.* 5-layer multiflask) at the identical other parameters (plating density, growth medium change frequency, and cultivation time), a comparison should also be done with caution. SD NCSCs showed a higher growth rate than HF NCSCs at P1, which is reflected by population doubling number (PDN) and population doubling time (PDT) values. The PDN was 6.04 ± 0.17 for SD NCSCs vs. 5.58 ± 0.24 for HF NCSCs (*p* < 0.01), and PDT was 27.86 ± 0.8 h for SD NCSCs vs. 30.13 ± 1.37 h for HF NCSCs (*p* < 0.01) (Table 1). These differences in the growth rate were retained at P2 and P3 under completely identical cell culture conditions (Table 1). Also, it should be noted that when harvesting confluent cultures at P2 and P3 SD NCSCs produced a higher average cell yield per 5-layer multiflask: 59.12 ± 4.29 × 10^6^ at P2 and 58 ± 4.84 × 10^6^ at P3 vs. 45.08 ± 4.52 × 10^6^ at P2 and 42.39 ± 1.74 × 10^6^ at P3 for HF NCSCs (*p* < 0.01) (Table 1). The approximate cell density in the confluent culture was 67400 cells per cm^2^ for SD NCSCs *vs.* 51400 cells per cm^2^ for HF NCSCs (*p* < 0.01). This difference can be allegedly explained by the smaller size of SD NCSCs compared to HF NCSCs. Moreover, any attempts to culture the dissected HFs from the skin within 3D fibrin hydrogel did not reveal any HF cell outgrowth (data not shown).

In addition to the higher growth rate of SD NCSCs at P3, they also showed a higher PE value: 34.1 ± 3.24% compared to 25.92 ± 4.5% of HF NCSCs (*p* < 0.01) (Table 1). At the same time, SD NCSCs and HF NCSCs demonstrated the similar capacity to self-renewal, which was assessed by subcloning of CFUs with the formation of secondary colonies (data not shown). Importantly, all HF and SD NCSC cultures after the large-scale expansion at P3 had a normal karyotype after GTG-banding assay (data not shown).

**Figure 3:**
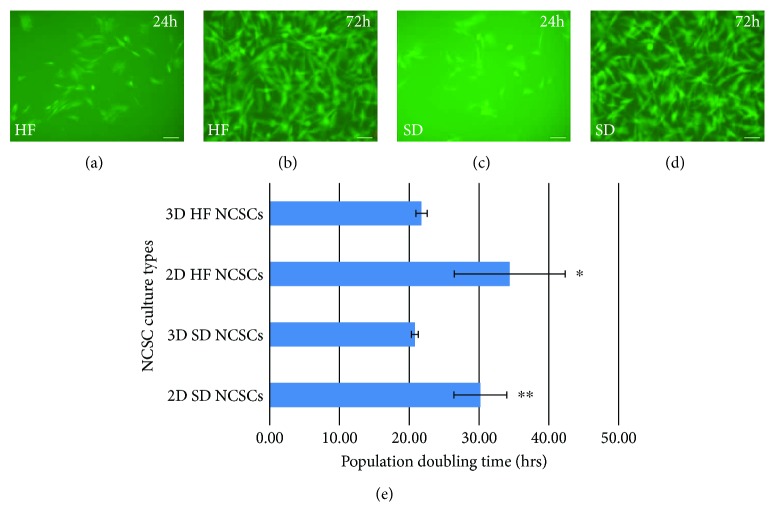
Three-dimensional growth of HF NCSC (a, b) and SD NCSC (c, d) in fibrin hydrogel. Fluorescein diacetate (FDA)/propidium iodide (PI) combined staining for live cells 24 h (a, c) and 72 h (b, d) after plating. (e) Diagram of the population doubling time (PDT) values for HF NCSCs and SD NCSCs populations grown in 2D vs. 3D cultures; initial plating: 10^4^ cells per 35 mm Petri dish; culture period: 168 h; mean ± SD for cultures from five donors. ^∗^ *p* < 0.05 between 2D and 3D HF; ^∗∗^ *p* < 0.01 between 2D and 3D SD.

However, to assess whether this difference in the growth rate of HF vs. SD NCSCs was due to the 3D cultivation step, we compared the growth rate of targeted NCSC populations in 2D vs. 3D cultures. We plated 10^4^ cultured cells from P3 per 35 mm Petri dish with (2.0 ml) or without fibrin hydrogel (*n* = 5). Both HF and SD NCSCs readily proliferated in 3D fibrin hydrogel as confirmed by fluorescein diacetate staining for live cells at 24 h and 72 h postplating (Figures 3(a)–3(d)). The cell culturing time was 168 h and the PDT values made 34.39 ± 7.95 h vs. 30.19 ± 3.79 h in 2D against 21.76 ± 0.8 h vs. 20.80 ± 0.49 h in 3D for HF and SD, respectively (Figure 3(e)). Evidently, 3D culture conditions revealed the lower PDT values for NCSC populations after culturing within 168 h. However, PDT values in 3D were comparable for both HF and SD NCSC, whereas in 2D culture system SD NCSCs demonstrated slightly higher proliferation rate.

Thus, in our culture conditions, SD NCSCs were characterized by significantly higher total cell yield number, growth rate, and clonogenic potential compared to HF NCSCs probably due to the primary 3D cultivation step.

### 3.3. Immunophenotype of Cultured NCSCs from Hair Follicles and Skin Dermis

**Figure 4:**
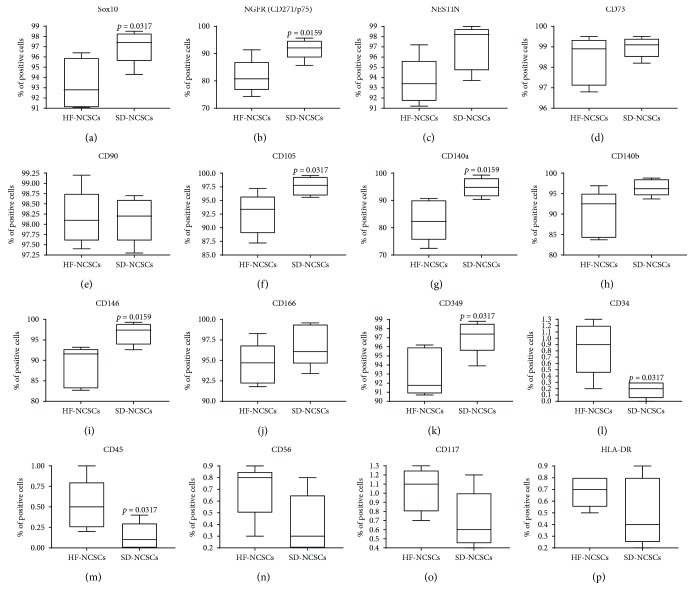
Immunophenotype analysis of NCSC cultures isolated from human hair follicles and skin dermis at P3. (a–p) Expression of characteristic NCSC markers by HF and SD NSCSs; mean ± SD for cultures from five donors.

**Figure 5:**
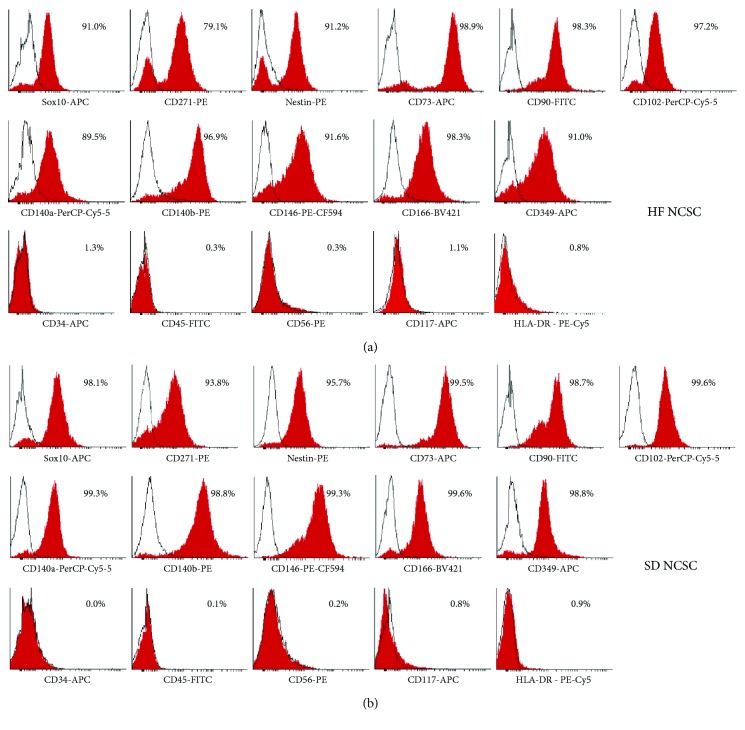
Representative FACS histograms from donor 1. (a) HF NCSCs. (b) SD NCSCs.

HF and SD NCSCs have similar phenotype and express the following markers at P3: CD271 (P75, LNGFR), SOX10 and NESTIN (markers of neural crest origin); CD73, CD90, and CD105 (multipotent mesenchymal stem/stromal cell markers); CD349 (FZD-9, receptor for growth factors of the WNT family), CD140a (PDGFR*α*), and CD140b (PDGFR*β*) (both are receptors for PDGFs); adhesion molecules CD146 (MCAM) and CD166 (ALCAM) (Figures 4 and 5; Table 2). Approximately, 7% SOX10^−^, 20% CD271^−^, and 10% CD105^−^ cells were discovered in cell cultures of adult HF NCSCs (Table 2). Almost all cells within HF NCSC and SD NCSC populations expressed CD73 and CD90. SOX10 and CD271 are known markers of adult NCSCs with neurogenic and gliogenic potential [10]. Thus, SOX10 maintains multipotency of NCSCs [49], but TGF*β*-mediated SOX10 suppression controls the generation of mesenchymal progenitors from NCSCs [50]. This may indicate the appearance of cell subpopulation with a differentiation potential limited only by mesenchymal cell fate. Moreover, due to known CD105 function [51], the cell subpopulation which is negative for this marker could have decreased osteogenic potential.

HF and SD NCSCs were negative for the following markers: CD34, CD45, and HLA-DR (hematopoietic and immune cells markers), CD56 (NCAM)—marker of mature neurons and glial cells; CD117 (C-KIT)—receptor for SCF, the marker of melanocytes and melanocyte stem cells. This indicates the lack of contamination of NCSC cultures by hematopoietic cells and any significant spontaneous differentiation into neurons, glial cells, and melanocytes during the large-scale expansion.

In a general manner, the population of SD NCSCs at P3 possesses a significantly greater cell number positive for the following markers: SOX10, CD271, CD105, CD140a, CD146, and CD349 compared to HF NCSC (*p* < 0.05).

### 3.4. Gene Expression Profiling in HF NCSC and SD NCSC Cultures

**Figure 6:**
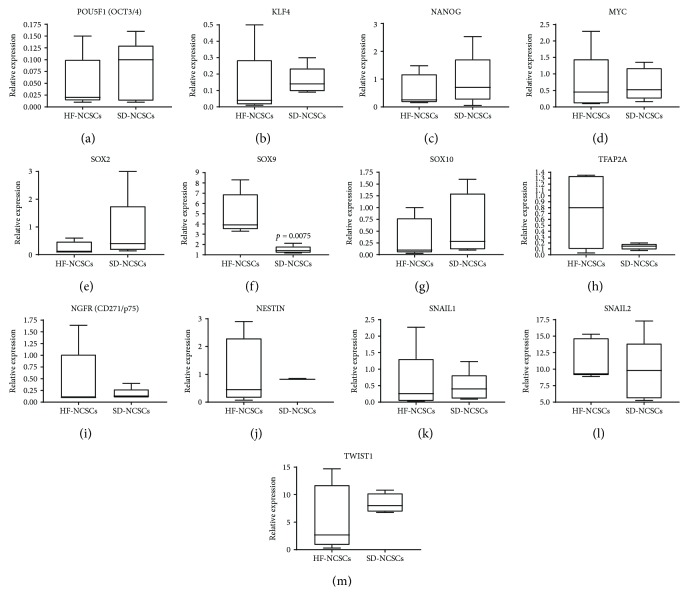
The mRNA expression levels of pluripotency, stemness, and neural crest markers for human NCSCs isolated from hair follicles and skin dermis; qPCR analysis. The expression of all target genes was normalised to *TBP* gene expression levels. The HF NCSC and SD NCSC cultures from five donors were analysed. Box plots show quartiles, median, minimum, and maximum values.

To obtain further insight into the molecular features of NCSCs isolated from HF and SD, we evaluated the mRNA expression level of the key genes linked with pluripotency, stemness, NC identity, and growth factors. HF NCSCs and SD NCSCs expressed at similar level the transcription factors *OCT3/4*, *SOX2*, *KLF4*, *C-MYC*, and *NANOG* that are essential for induction and maintenance of pluripotent state during the embryonic development and iPS/ES generation [52] (Figures 6(a)–6(e)). At the same time, mRNA of *LIN28A*, which is involved in the regulation of pluripotency, reprogramming, and stem cell metabolism [53], was not detected neither in HF NCSCs nor in SD NCSCs. The mRNA expression of NC identity markers was assessed to prove the origin of cell cultures isolated from HF and SD. All analysed HF NCSC and SD NCSC cultures expressed the key NC identity markers, such as *SOX9*, *SOX10*, *TFAP2A*, *LNGFR (CD271/P75), NESTIN*, *SNAIL1*, *SNAIL2 (SLUG)*, and *TWIST1* (Figures 6(f)–6(m)). Significantly elevated mRNA level of *SOX9* was specific for HF NCSCs (Figure 6(f)). It should be noted that *SOX9*, *TFAP2A*, *NGFR (CD271/p75)*, *NESTIN*, and *TWIST1* were more uniformly expressed in SD NCSCs compared to HF NCSCs (Figures 6(f), 6(h)–6(j), and 6(m)). This indicates that the isolation and expansion of NCSCs from SD using new methodological approach allows obtaining more homogeneous cell population with peculiar phenotype features of adult NCSCs.

**Figure 7:**
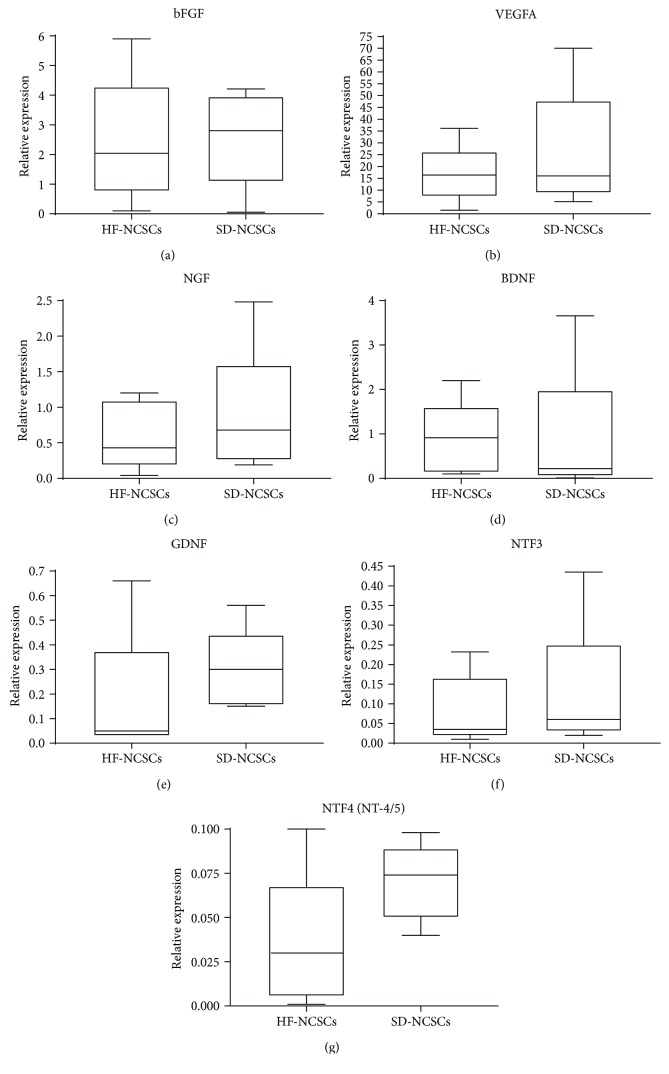
The mRNA expression levels of growth factors in human NCSC cultures isolated from hair follicles and skin dermis; qPCR analysis. The expression of all target genes was normalised to *TBP* gene expression levels. The HF NCSC and SD NCSC cultures from five donors were analysed. Box plots show quartiles, median, minimum, and maximum values.

Numerous cytokines and growth factors are involved in maintaining stem cell self-renewal property, regulation of cell fate, and mediating stem cell therapeutic potential. High levels of *VEGFA* and *FGF2* mRNA were detected in NCSCs isolated from HF and dermis (Figures 7(a) and 7(b)). FGF-2 growth factor is essential for cell survival and proliferation, while VEGF-A protein induces angiogenesis that is crucial for successful tissue regeneration. HF NCSCs and SD NCSCs express similar mRNA levels of neurotrophins required for nerve and glial cell survival—*NGF*, *BDNF*, *GDNF*, *NTF3*, and *NTF4/5* (Figures 7(c)–7(g)). However, mRNA of *LIF* was not detected neither in HF NCSCs nor in SD NCSCs (data not shown).

Accordingly, HF NCSCs and SD NCSCs expressed *OCT3/4*, *KLF4*, *NANOG*, *C-MYC*, and *SOX2* pluripotency markers at the mRNA level. Based on NC identity marker expression pattern, cell cultures isolated from HF and dermis demonstrated NC origin. Both HF and SD NCSCs express the key cell survival, angiogenic, neurotrophic, and proliferation promoting growth factors, such as *FGF2*, *VEGFA*, *NGF*, *BDNF*, *GDNF*, NTF3, and *NTF4/5*.

### 3.5. Growth Factors and Cytokines Secretion Patterns by HF NCSCs and SD NCSCs

**Figure 8:**
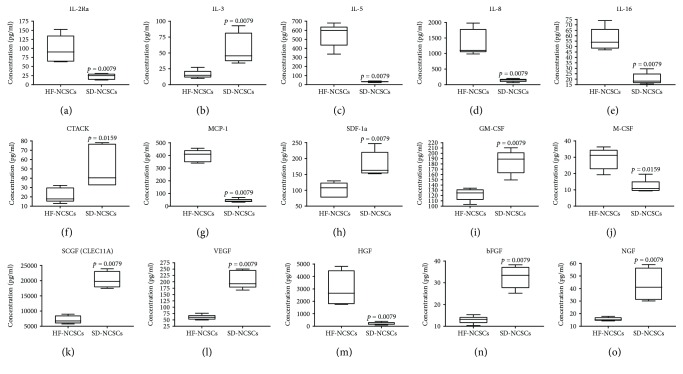
Secretion patterns of chemokines, cytokines, and growth factors by adult NCSCs from hair follicle and skin dermis.

Secretion levels of chemokines, cytokines, and growth factors by HF and SD NCSCs were determined using 27-Plex and 21-Plex Bio-Plex^®^ multiplex immunoassay. HF and SD NCSCs secreted 15 proteins from a kit of 48 chemokines, cytokines, and growth factors that were analysed (Figure 8). Despite the common qualitative spectrum of the secreted proteins, a statistically significant difference in the quantitative production between HF and SD NCSCs was established. HF NCSCs produced more proinflammatory IL-6 and IL-8 cytokines, IL-16 pleiotropic cytokine, soluble IL-2 receptor alpha chain (IL-2Ra), MCP-1 chemokine, M-CSF, and HGF growth factors (*p* < 0.01). On the other hand, SD NCSCs produced more IL-3 cytokine, CTACK and SDF-1a chemokines, GM-CSF, SCGF, VEGF-A, FGF-2, and NGF growth factors. Interestingly, despite significantly higher VEGF-A, FGF-2, and NGF protein production by SD NCSCs, the mRNA expression of the corresponding genes did not show a statistically significant difference.

### 3.6. Directed Multilineage Differentiation and Sphere-Forming Capacity of Adult HF NCSCs and SD NCSCs

For further confirmation of the NC origin and assessment of the functional properties of adult HF and SD NCSCs, we performed a study of their ability to grow as neurosphere-like structures and to realize the directed multilineage differentiation into the peculiar cell types—the NC derivatives.

**Figure 9:**
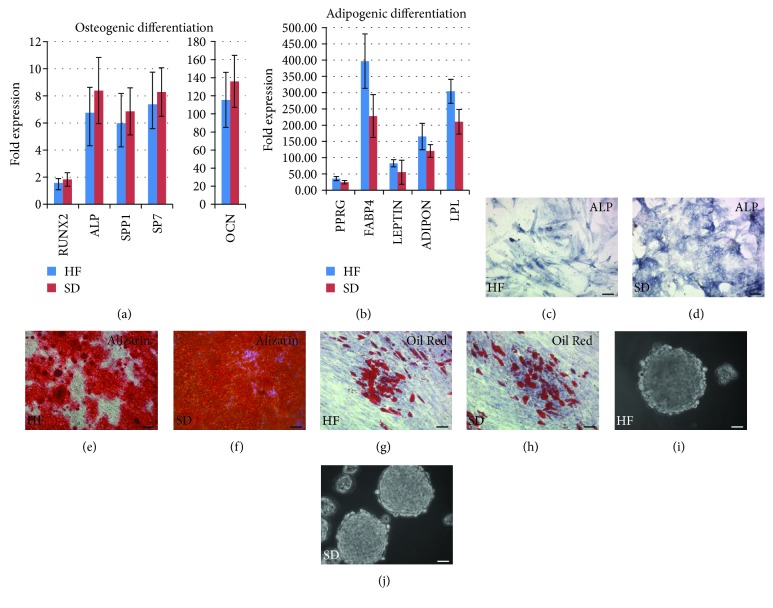
Mesenchymal differentiation and neurosphere-like structure formation of HF (c, e, g, i) and SD NCSC cultures (d, f, h, j) at P3. (a) Osteogenic differentiation, 14 days, qPCR analysis. (b) Adipogenic differentiation, 14 days, qPCR analysis. The expression of all target genes was normalised to *TBP* gene expression levels and then to the undifferentiated control cells. The HF NCSC and SD NCSC cultures from five donors were analysed. (c, d) Osteogenic differentiation, 10 days, stain with BCIP^®^/NBT Liquid Substrate System for ALP activity (dark blue). (e, f) Osteogenic differentiation, 21 days, Alizarin Red S stain for mineralized extracellular matrix deposits (red). (g, h) Adipogenic differentiation, Oil Red O stain for neutral lipid vacuoles (red) and Romanowsky-Giemsa counterstain. (i, j) Neurosphere-like structures, phase-contrast microscopy. (c–h) Scale bar: 100 *μ*m. (i, j) Scale bar: 50 *μ*m.

**Figure 10:**
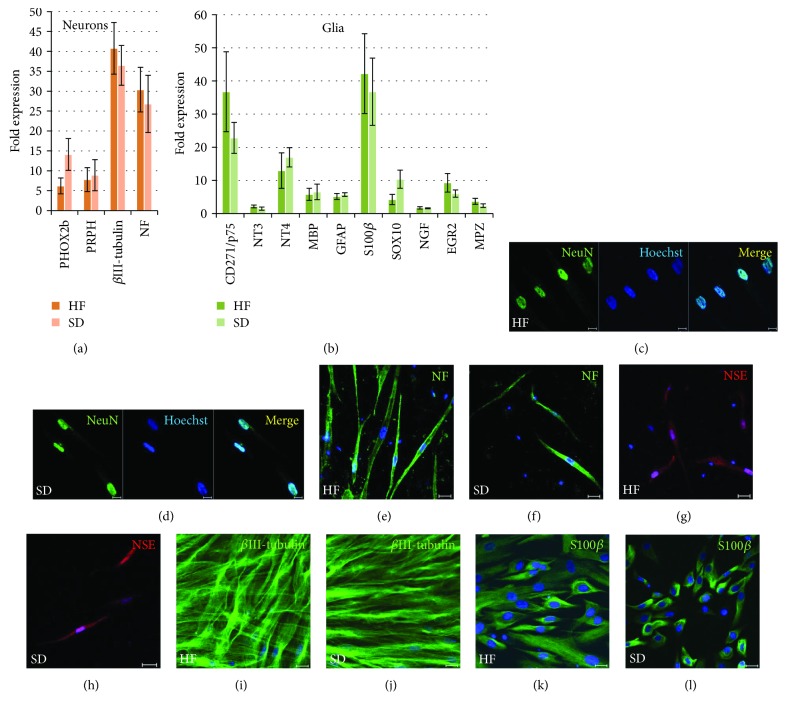
Neuronal and glial differentiation of HF NCSCs and SD NCSCs. mRNA expression levels of neuronal (a) and glial (b) markers; qPCR analysis. The expression of all target genes was normalised to *TBP* gene expression levels and then to the undifferentiated control cells. The HF NCSC and SD NCSC cultures from five donors were analysed. (c–j) Immunocytochemical analysis of neuronal and glial (Schwann cells). (k, l) Marker expression by HF NCSCs (c, e, g, i, k) and SD NCSCs (d, f, h, j, l). (c, d) NeuN (green); (e, f) neurofilament (NF; green); (g, h) neuron-specific enolase (NSE; red); (i, j) *β*III-tubulin (green); (k, l) S100*β* (green). The nuclei were stained with Hoechst 33342 dye; confocal microscopy. (c, d) Scale bar: 10 *μ*m. (e–l) Scale bar: 20 *μ*m.

The results of the HF and SD NCSC differentiation into mesenchymal cell lineages (adipocytes and osteoblasts) are presented in Figures 9(a)–9(h). Under specific serum-free conditions, HF and SD NCSCs demonstrated an ability to grow as neurosphere-like structures (Figures 9(i) and 9(j)). Also, human adult cultured HF and SD NCSCs were able to differentiate into neural (neurons and Schwann cells) cell types (Figure 10) that is a characteristic for cranial NC origin. Both cultures after differentiation express key neuronal markers such as *PHOX2b*, *peripherin (PRPH)*, *βIII-tubulin*, and *neurofilament* at the mRNA level as well as NeuN, neuron-specific enolase, *β*III-tubulin, and neurofilament at the protein level. As for the glial differentiation, HF and SD NCSCs express *p75(CD271)*, *neurotrophins 3* and *4 (NT-3* and *NT-4*), *myelin basic protein (MBP)*, *glial fibrillary acidic protein (GFAP)*, *SOX10*, *nerve growth factor (NGF)*, *early growth response 2 (EGR2)*, and *myelin protein zero(MPZ)* at the mRNA level and S100*β* both at the mRNA and protein levels.

## 4. Discussion

The HF and SD are well-known sources of adult NCSCs. There are several niches containing adult NCSCs in the HF. Thus, Sieber-Blum and coauthors have shown the existence of NCSCs in the HF bulge region and originally named them as “epidermal neural crest stem cells” (EPI-NCSCs) [8]. At the same time Fernandes with coauthors [9] identified the dermal papillae of HF as a niche for the previously described “skin-derived precursor cells” (SKPs) and demonstrated by cell lineage tracing analysis their NC origin. Later, SKPs were isolated from human neonatal foreskin, which does not have HFs [43]. In parallel, Wong and coauthors isolated the multipotent stem cells of NC origin from the adult face and body skin of mice and humans [10] based on their sphere-forming capacity. These multipotent stem cells expressed Sox10 and p75 antigens, showed extensive self-renewal under sphere culture conditions, and had the ability to undergo directed multilineage differentiation into neurons, glial cells, melanocytes, smooth muscle cells, adipocytes, osteoblasts, and chondrocytes [21, 22]. Several sources of NCSCs within the face skin were revealed using cell fate mapping analysis: many mesenchymal structures of HF (capsula, dermal sheath, and dermal papillae), glial cell lineage linked with nerve ending, and melanocyte cell lineage. At the same time, the sphere-forming NCSCs from the back skin were only of glial and melanocyte cell lineages.

Several studies describe the production and large-scale expansion of adult NCSCs from human HFs by explant technique [44, 45]. All authors note the following drawbacks of this technique: high frequency of explant detachment, spontaneous outgrowth of keratinocytes from explants of HFs, and long-term primary culture step to obtain a sufficient number of cells for subsequent passaging. Our method for isolation and large-scale expansion of NCSCs from the SD is based on cell population preplating followed with cell growth within fibrin hydrogel. To avoid the drawbacks that are presented in explant method and neurosphere-forming approach, we used the preplating technique as a selection method with the transfer of a nonadherent cell population into 3D PDFH for primary cultivation. Previously, PDFH was used to expand human adult NCSCs from nasal inferior turbinate after their isolation on the basis of sphere-forming capacity [47]. Importantly, our group and Greiner with coauthors [47] used various methods for the preparation of PDFH. To create PDFH by Greiner et al., 10% blood plasma was added to the total serum-free growth medium, whereas in our protocol, 10% blood serum supplemented with 10% CaCl_2_ was added to the blood plasma and the growth medium was next added to the T25 flask after 3D hydrogel polymerization. Thus, in these two protocols, the amount of fibrinogen used to prepare 3D fibrin hydrogel differs by approximately 10-fold and leads to the formation of hydrogels with different stiffness and density of the fibrin fiber network. It is well known that the stiffness of a hydrogel can have great impact on the properties of cultured cells [54]. In further studies, it would be important to compare the growth rate, phenotype, and biological properties of adult NCSCs after their expansion within 3D fibrin hydrogels prepared according to these two different protocols. It should be noted that PDFH can be used for large-scale expansion of adult NCSCs from different tissue sources. Moreover, the 3D culture system proposed here can be animal serum-free and subsequently could be developed into completely xeno-free system, which reflects the actual requirements of many regulatory bodies for the production of human cell-based medicinal products without substances of xenogeneic provenance.

The adult SD NCSCs and HF NCSCs obtained after the primary culture had identical expression pattern of characteristic markers (SOX10, CD271, NESTIN, SOX2, and CD349) but possessed slightly different cell morphology. Further expansion showed a faster growth rate of SD NCSCs compared to HF NCSCs in the standard 2D cell culture system starting from P1 to P3 (final passage in our study). Theoretically, using our original isolation and large-scale expansion method, the therapeutic dose of SD NCSCs (100-300 million cells) can already be obtained at P1 after three weeks of culturing time in total. For HF NCSCs, an additional passage is required before proceeding to the cell culture flasks with a large growth surface area, which requires one more additional week of culturing.

In general, the resultant populations of SD NCSCs and HF NCSCs obtained after the large-scale expansion had the same immunophenotype, SOX10^+^CD271^+^Nestin^+^CD73^+^CD90^+^CD105^+^CD140a^+^CD140b^+^CD146^+^CD166^+^CD349^+^CD34^−^ CD45^−^ CD56^−^ CD117^−^HLA-DR^−^, showed a similar level of gene expression, produced the same spectrum of chemokines, cytokines, and growth factors, and also had the ability to undergo directed multilineage differentiation and formation of neurosphere-like structures. However, in addition to the variable growth rate, between the populations of SD NCSCs and HF NCSCs, there were also other significant differences at the final passage that could affect the potency of the cell-based medicinal products. These differences include the number of CFUs and the content of SOX10^+^, CD271^+^, and CD105^+^ cells. The number of CFUs has a direct relationship with the proliferative and reparative potential of the cell population. SOX10 and CD271 are common markers for multipotent adult NCSCs [10]. At the same time, SOX10 is the key transcription factor that determines the multipotency of NCSCs [49]. Concerning CD105, it is shown that its expression is characteristic for the subpopulations of human adipose-derived stem cells and stem cells from human exfoliated deciduous teeth (SHED) with an increased osteogenic potential [51]. The greater osteogenic potential of CD105^+^ cells can be explained by the functional role of CD105 (endoglin) as an additional coreceptor for numerous growth factors from TGF*β*/BMP superfamily [55]. It should be noted that SHED originated from the NC [56], but the relationship of CD105 to the osteogenic potential is not indicated for NC cells from other tissue sources. It is also known that during embryonic development, CD105 is required for myogenic differentiation of early NCSCs into the smooth muscle cells [57]. The myogenic differentiation potential of adult HF and SD NCSCs has not been tested and requires further experimental confirmation. The verification of the relationship between CD105 and the osteogenic potential of adult HF and SD NCSCs is also a promising area for future studies. Regarding CD140a, CD146, and CD349, further research is needed to clarify their role in the biology of adult NCSCs, but potentially, they can be associated with gliogenesis, migration, and proliferation and thus also can strongly affect the potency of adult NCSC-based medicinal products.

Adult HF and SD NCSCs exhibited a similar gene expression profiling, with the exception of the *SOX9* mRNA expression level. In our study, we did not detect SOX9 at the protein level and did not determine the number of SOX9^+^ cells in the final populations of adult HF and SD NCSCs. These are important issues for future study, together with the definition of the SOX9 role in the biology of adult HF and SD NCSCs. In general, it should be noted that SD NCSC cultures demonstrated more homogeneous gene and surface antigen expression patterns compared to HF NCSCs, as assessed by qPCR and FACS, which indicates better reproducibility of the SD NCSC isolation and large-scale expansion preplating/3D fibrin hydrogel method in contrast to the HF explant method.

Regarding the profile of secreted chemokines, cytokines, and growth factors, SD NCSCs look more preferable than HF NCSCs for use in regenerative medicine due to lower secretion of proinflammatory cytokines IL-6 and IL-8 b and greater secretion of widely known regulators of reparative regeneration, such as VEGF-A, NGF, FGF-2, and SDF-1a, which orchestrate neoangiogenesis, nerve fiber growth, proliferation, and homing of various types of stem/progenitor cells, respectively.

At the moment, it is not possible to say unambiguously whether the observed biological differences between the populations of adult HF and SD NCSCs (evaluated here via cell growth rate, phenotype profile, gene expression, chemokines/cytokines/growth factors production, and the number of CFUs in the final cell-based product) are their intrinsic properties, or they appear due to the previous SD NCSC culturing step in PDFH. Theoretically, culturing within PDFH can affect the properties of adult NCSCs, diverting them towards the enhancing of reparative potential by simulating a wound bed microenvironment. An important and intriguing goal of future research is to compare the effectiveness of the large-scale expansion of HF and SD NCSCs in the standard 2D and 3D neurosphere-like cell culture system vs. PDFH culturing and to assess the biological properties of the resultant cell populations. The subsequent progress in large-scale expansion methods of human adult HF and SD NCSCs for clinical applications is also related to the development of completely xeno-free cell culture protocols.

Summing up, we have demonstrated that HF and particularly SD are suitable sources for the large-scale production of human adult NCSCs with similar properties. At the same time, the protocol of isolation and expansion of adult NCSCs from SD within PDFH has significant advantages in terms of timing interval required for obtaining cell therapeutic dose with high content of clonogenic cells (by CFU assay) within the final cell-based medicinal product. Moreover, the resultant population of SD NCSCs has a higher content of SOX10, CD271, and CD105 positive cells and reveals more homogenous profile of gene expression and more preferable spectrum of produced growth factors, cytokines, and chemokines.

## 5. Conclusions

We have compared the biological properties of human adult HF NCSCs isolated via explant method with SD NCSCs isolated via preplating/3D fibrin hydrogel growth method. Both populations demonstrated expression of robust neural crest origin markers, such as SOX10, CD271, NESTIN, SOX2, and CD349. They also had similar gene expression profiling, SOX10^+^CD271^+^Nestin^+^CD73^+^CD90^+^CD105^+^CD140a^+^CD140b^+^CD146^+^CD166^+^CD349^+^CD34^−^ CD45^−^ CD56^−^ CD117^−^HLA-DR^−^ immunophenotype and were able to undergo directed multilineage differentiation into mesenchymal (osteo, adipo) and neural (neurons, glia) cell types. However, they demonstrated significantly different cytokine expression levels. Interestingly, SD NCSC demonstrated higher proliferation rate and formed more homogenous population during large-scale expansion. Together these data make large-scale expended SD NCSCs an attractive cell type for potential various clinical applications.

## Figures and Tables

**Table 1:** Growth parameters of adult NCSCs cultures originated from human hair follicles and skin dermis.

Cell line	P0		P1				P2				P3				CFU frequency at P3 (%)
	Cell culture vessel	Cell yield (×10^6^)	Cell culture vessel	Cell yield (×10^6)^	PDN	PDT (hours)	Cell culture vessel	Cell yield (×10^6^)	PDN	PDT (hours)	Cell culture vessel	Cell yield (×10^6^)	PDN	PDT (hours)	
HF NCSCs 1	2 × Petri dish, ø 35 mm	0.183	T25 flask	1.355	5.74	29.27	5-layer multiflask	44.394	5.64	29.79	5-layer multiflask	42.155	5.57	30.16	22.4
HF NCSCs 2	2 × Petri dish, ø 35 mm	0.142	T25 flask	0.93	5.20	32.31	5-layer multiflask	39.462	5.46	30.77	5-layer multiflask	41.647	5.55	30.27	26.8
HF NCSCs 3	2 × Petri dish, ø 35 mm	0.225	T25 flask	1.265	5.64	29.79	5-layer multiflask	42.727	5.59	30.05	5-layer multiflask	40.032	5.5	30.55	20.5
HF NCSCs 4	2 × Petri dish, ø 35 mm	0.114	T25 flask	1.428	5.81	28.92	5-layer multiflask	51.215	5.85	28.72	5-layer multiflask	44.509	5.65	29.73	31.7
HF NCSCs 5	2 × Petri dish, ø 35 mm	0.163	T25 flask	1.173	5.53	30.38	5-layer multiflask	47.605	5.75	29.22	5-layer multiflask	43.584	5.62	29.89	28.2

*Mean*		**0.165**		**1.230**	**5.584**	**30.132**		**45.081**	**5.658**	**29.709**		**42.385**	**5.578**	**30.121**	**25.92**
*SD*		*0.042*		*0.193*	*0.239*	*1.336*		*4.516*	*0.150*	*0.786*		*1.738*	*0.059*	*0.318*	*4.50*
SD NCSCs 1	T25 flask 3D gel	2.192	5-layer multiflask	56.284	5.99	28.05	5-layer multiflask	58.544	6.04	27.81	5-layer multiflask	53.057	5.90	28.47	31.5
SD NCSCs 2	T25 flask 3D gel	2.517	5-layer multiflask	59.318	6.06	27.72	5-layer multiflask	62.05	6.13	27.41	5-layer multiflask	60.827	6.10	27.54	33.2
SD NCSCs 3	T25 flask 3D gel	3.207	5-layer multiflask	66.436	6.22	27.01	5-layer multiflask	64.719	6.19	27.14	5-layer multiflask	62.586	6.14	27.36	38.4
SD NCSCs 4	T25 flask 3D gel	1.458	5-layer multiflask	48.375	5.77	29.12	5-layer multiflask	54.894	5.95	28.24	5-layer multiflask	52.455	5.88	28.57	30.9
SD NCSCs 5	T25 flask 3D gel	2.372	5-layer multiflask	62.085	6.13	27.41	5-layer multiflask	55.325	5.96	28.19	5-layer multiflask	61.095	6.10	27.54	36.5

*Mean*		**2.349**		**58.500**	**6.034**	**27.860**		**59.106**	**6.054**	**27.757**		**58.004**	**6.024**	**27.898**	**34.10**
*SD*		*0.629*		*6.781*	*0.170*	*0.800*		*4.258*	*0.105*	*0.480*		*4.842*	*0.124*	*0.576*	*3.24*
		*p* < 0.001			*p* < 0.01	*p* < 0.01		*p* < 0.01	*p* < 0.01	*p* < 0.01		*p* < 0.01	*p* < 0.001	*p* < 0.001	*p* < 0.01

**Table 2:** Phenotype characterization of adult NCSC cultures originated from human hair follicles and skin dermis at passage 3; flow cytometry.

Cell line	Antigens															
	Sox10	CD271	Nestin	CD73	CD90	CD105	CD140a	CD140b	CD146	CD166	CD349	CD34	CD45	CD56	CD117	HLA-DR
HF NCSCs 1	91.0	79.1	91.2	98.9	98.3	97.2	89.5	96.9	91.6	98.3	91.0	1.3	0.3	0.3	1.1	0.8
HF NCSCs 2	96.4	82.5	97.2	99.2	98.1	94.3	82.3	92.5	93.2	95.4	95.7	0.9	0.5	0.8	0.9	0.6
HF NCSCs 3	92.8	80.8	93.4	97.4	97.8	90.8	72.4	83.7	82.7	91.8	91.6	0.2	0.6	0.9	1.3	0.7
HF NCSCs 4	95.4	91.4	92.2	99.5	99.2	93.4	90.7	93.1	92.4	94.7	96.2	0.7	0.2	0.7	0.7	0.8
HF NCSCs 5	91.2	74.3	94.1	96.8	97.4	87.2	78.7	84.6	83.6	92.5	90.7	1.1	1.0	0.8	1.2	0.5

*Mean*	**93.36**	**81.62**	**93.62**	**98.36**	**98.16**	**92.58**	**82.72**	**90.16**	**88.70**	**94.54**	**93.04**	**0.84**	**0.52**	**0.70**	**1.04**	**0.68**
*SD*	*2.45*	*6.27*	*2.29*	*1.19*	*0.67*	*3.78*	*7.62*	*5.75*	*5.11*	*2.58*	*2.68*	*0.42*	*0.31*	*0.23*	*0.24*	*0.13*
SD NCSCs 1	98.1	93.8	95.7	99.5	98.7	99.6	99.3	98.8	99.3	99.6	98.8	0.0	0.1	0.2	0.8	0.9
SD NCSCs 2	97.4	92.1	98.2	98.8	97.9	95.6	90.4	93.7	95.1	95.8	97.2	0.3	0.0	0.3	1.2	0.2
SD NCSCs 3	98.5	95.7	99.0	99.3	97.3	97.8	94.8	96.2	98.6	96.1	98.3	0.1	0.0	0.8	0.4	0.4
SD NCSCs 4	94.3	85.7	93.7	98.2	98.2	96.2	92.5	95.4	92.6	93.4	93.9	0.2	0.4	0.2	0.5	0.7
SD NCSCs 5	96.9	91.4	98.5	99.1	98.5	99.1	97.1	98.3	97.4	99.2	97.4	0.3	0.2	0.5	0.6	0.3

*Mean*	**97.04**	**91.74**	**97.02**	**98.98**	**98.12**	**97.66**	**94.82**	**96.48**	**96.60**	**96.82**	**97.12**	**0.18**	**0.14**	**0.40**	**0.70**	**0.50**
*SD*	*1.65*	*3.76*	*2.25*	*0.51*	*0.55*	*1.75*	*3.54*	*2.10*	*2.75*	*2.58*	*1.91*	*0.13*	*0.17*	*0.25*	*0.32*	*0.29*
	*p* < 0.05	*p* < 0.05				*p* < 0.05	*p* < 0.05		*p* < 0.05		*p* < 0.05					

## Data Availability

The data used to support the findings of this study are available from the corresponding author upon request.

## Abbreviations

ALP: Alkaline phosphatase
CFUs: Colony-forming units
EPI-NCSCs: Epidermal neural crest stem cells
FDA: Fluorescein diacetate
HF: Hair follicle
HF NCSCs: Hair follicle-derived NCSCs
NAMS: National Academy of Medical Sciences
NC: Neural crest
NCSCs: Neural crest-derived stem cells
P3: Cell culture passage number, e.g., passage 3
PDFH: 3D blood plasma-derived fibrin hydrogel
PDN: Population doubling number
PDT: Population doubling time
PE: Plating efficiency
PI: Propidium iodide
SD: Skin dermis
SD NCSCs: Skin dermis-derived NCSCs
SHED: Stem cells from human exfoliated deciduous teeth
SKPs: Skin-derived precursor cells.

## Supplementary Materials

Supplementary Materials Table S1: list of antibodies used in this study for the identification of human cultured hair follicle and skin dermis-derived adult NCSCs and their differentiated derivative cell types. Table S2: PCR primer sequences used in this study for the identification of human cultured hair follicle- and skin dermis-derived adult NCSCs and their differentiated derivative cell types.
