# Supplementary material for: Comparative Analysis of Biological Properties of Large-Scale Expanded Adult Neural Crest-Derived Stem Cells Isolated from Human Hair Follicle and Skin Dermis
Source: Stem Cells Int. 2019 Feb 19;2019:9640790. doi: 10.1155/2019/9640790 (PMC6399535; doi:10.1155/2019/9640790)
Supplement: Supplementary Materials — Table S1: list of antibodies used in this study for the identification of human cultured hair follicle and skin dermis-derived adult NCSCs and their differentiated derivative cell types. Table S2: PCR primer sequences used in this study for the identification of human cultured hair follicle- and skin dermis-derived adult NCSCs and their differentiated derivative cell types. [file 9640790.f1.pdf]

Supplementary materials

Supplementary Table 1. Antibodies used in a study

Antigen	Fluorochrome	Host	Clone	Manufacturer
CD105	PerCP-Cy5.5	mouse	266	BD Pharmingen™
CD117	APC	mouse	YB5.B8	BD Pharmingen™
CD140a	PerCP-Cy5.5	mouse	αR1	BD Pharmingen™
CD140b	PE	mouse	28D4	BD Pharmingen™
CD146	PE-CF594	mouse	P1H12	BD Horizon™
CD166	BV421	mouse	3A6	BD Horizon™
CD34	APC	mouse	581	BD Pharmingen™
CD349	Unconjugated	rabbit	Polyclonal	Bioss
CD45	FITC	mouse	HI30	BD Pharmingen™
CD56	PE	mouse	B159	BD Pharmingen™
CD73	APC	mouse	AD2	BD Pharmingen™
CD90	FITC	mouse	5E10	BD Pharmingen™
HLA-DR	PE-Cy5	mouse	G46-6	BD Pharmingen™
Nestin	Unconjugated	rabbit	Polyclonal	Sigma-Aldrich
Nestin	PE	mouse	25/NESTIN	BD Pharmingen™
NeuN	Unconjugated	mouse	377	Chemicon, Millipore
NF (NF-H)	Unconjugated	mouse	RT97	DSHB
NSE	Unconjugated	rabbit	Polyclonal	Bioss
p75 (CD271)	Unconjugated	rabbit	Polyclonal	BioLegend
p75 (CD271)	PE	mouse	C40-1457	BD Pharmingen™
S100β	Unconjugated	rabbit	Polyclonal	Abcam
Sox10	Unconjugated	mouse	20B7	R&D Systems
Sox10	Unconjugated	mouse	SOX10/991 +	Novus Biologicals

			SOX10/1074	
Sox2	Unconjugated	rabbit	Polyclonal	BioLegend
βIII-tubulin	Unconjugated	mouse	SDL.3D10	Sigma-Aldrich

Table S1 – List of antibodies used in this study for identification of human cultured hair follicle- and skin dermis-derived adult NCSCs and their differentiated derivative cell types.

Supplementary Table 2. The primers sequence used in a study

Gene	**Forward primer 5'→3'**	**Reverse primer 5'→3'**
TBP	ccactcacagactctcacaac	ctgcggtacaatcccagaact
POU5F1(OCT3/4)	tgtactcctcggtccctttc	tccaggttttctttccctagc
TERT	ctccatcctgaaagccaagaa	agtcagcttgagcaggaatg
LIN28	agcctcatgtccgctgaagg	gcagggtagggctgtggatt
KLF4	tcccgccgctccattaccaa	ggggtgaagaaggtggggtga
NANOG	tcccctcctcccatccctca	ggctccaaccatactccaccct
MYC	agaacggagggagggatcgc	ctcgctccctctgcctctcg
SOX2	cagctcgcagacctacatga	tggagtgggaggaagaggta
SOX9	gaaagagaggaccaaccagaat	ttgggtacgagttgcctttag
SOX10	tttgactactctgaccatcagccc	agtgtcgtatatactggctgctcc
TFAP2A	cgaaaccgaatttcctgccaaagc	agatgaggttgaagtgggtcaagc
NGFR (CD271/p75)	acctccagaacaagacctcatagc	ttgttctgcttgcagctgttcc
NESTIN	agaggaagagaacctgggaaag	ttggtccttctccaccgtatc
SNAIL1	ccacgaggtgtgactaactatg	accaaacaggaggctgaaata
SNAIL2	cctgtgctgaccaaccaaata	atgctcttgcagctctctct
TWIST1	cgggagtccgcagtctta	gcttgagggtctgaatcttg
FGF2	caccagtggatagtgtgagaga	gccaatcttggtttcaggtactta
VEGFA	ccatcgacagaacagtccttaatc	tggcgaatccaattccaagag
NGF	taccaagggagcagctttcta	cattgctctctgagtgtggttc
BDNF	gtcaagttgggagcctgaaatag	aggtgtgaaatgggctgaatg
GDNF	acttgggtctgggctatgaaac	ctttgtcactcaccagccttct
NTF3	ggcgcaactactttcttctctct	agctcggtcattcaaagtctcc
NTF4/5	tcctatgtgcgggcattga	tctcagcatccagctctgttatt

LIF	gaagggtcggatctgagagaat	ctggaccctgacaccctaaa

Table S2 – PCR primer sequences used in this study for identification of human cultured hair follicle- and skin dermis-derived adult NCSCs and their differentiated derivative cell types.
